# Are adults with autism receiving regular preventive dental services?

**DOI:** 10.1111/scd.12738

**Published:** 2022-05-30

**Authors:** Robin McNeil, Kimberly Krust Bray, Tanya Villalpando Mitchell, Chandler Pendleton, Leonardo Marchini

**Affiliations:** ^1^ The University of Iowa College of Dentistry and Dental Clinics Iowa City Iowa USA; ^2^ School of Dentistry University of Missouri‐Kansas City Kansas City Missouri USA

**Keywords:** adult, autism spectrum disorder, autistic disorder, dental care for disabled, dental care

## Abstract

**Purpose/Aim:**

To investigate the frequency of preventive dental care among adults with autism and explore factors associated with receiving regular preventive care.

**Materials and Methods:**

De‐identified data was collected from electronic health records of 18‐year‐old or older patients with autism that had at least one preventive dental procedure recorded. The data was then analyzed to describe the frequency of preventive dental procedures provided for this population and investigate what variables are associated with regular care.

**Results:**

Sample size was 119, 67% were males, average age was 30.8 years, and 58% had Medicaid. Average BMI was 42.8, the prevalence of diabetes and heart disease were 16% and 34%, respectively, and 86% reported mental health problems. Recreational drug use was 6.8%, alcohol use was 19%, and tobacco use 16%. Xerostomia was reported by 32%, and the average number of medications was 7.2 ± 5.5. The average number of preventive dental visits was 7.9 ± 10.6, and 35% of the patients had at least one preventive dental visit per year. Only number of medications had a statistically significant association with number of preventive dental visits.

**Conclusions:**

Only one in every three adults with autism had at least one preventive dental visit per year.

## 1 INTRODUCTION

Autism spectrum disorder (ASD) is a complex and pervasive neurodevelopmental condition that can include communication deficits, behavioral issues, and intellectual impairment.^1^, ^2^ As a consequence, ASD severely impacts social development.^1^, ^2^ ASD is more common in males than in females^2^ and it is usually diagnosed between the ages of 2 and 11 years of age.^3^ It is a prevalent condition, and current research shows that ASD affects approximately 2.5% of the US children and adolescents.^4^

For persons with ASD, accessing dental care requires overcoming many obstacles, including communicating effectively with care providers, obtaining consent from guardians, and sensory challenges in a new environment, and these obstacles may prevent a person from being able to effectively cope with receiving dental care.^5^, ^6^, ^7^, ^8^ Careful consideration of the needs and barriers related to ASD can help dental practitioners personalize their treatment plans.^6^, ^7^ However, many dental practitioners are not knowledgeable about the special needs for individuals with ASD and may feel uncomfortable when providing dental care for these patients.^9^, ^10^ This lack of confidence may be, in part, caused by a lack of research‐derived data about the dental needs of persons with ASD.^10^, ^11^, ^12^

There is currently more empirical evidence to support appropriate medical and dental care for children with ASD^13^ but limited research regarding dental care for adults with ASD.^11^, ^12^ The lack of data precludes a more comprehensive understanding of the dental needs of adults with autism. As a consequence, specific, customized strategies for providing dental care for adults with autism still need to be developed,^14^ and the lack of such protocols may reduce the confidence of practitioners in providing dental care for these patients and limit the effectiveness of treating persons with ASD. Considering the lack of research‐based evidence available to guide dental care provision for the adult patients with autism, more investigative efforts are necessary to understand the barriers for adults with autism to access dental care and what is needed to enable them to overcome those barriers.

This study will investigate the frequency of preventive dental care among adults with autism and explore what factors are associated with frequent preventive care. Clarifying the frequency and what variables are associated with regular preventive dental care can help identifying barriers and enable practitioners to improve access to preventive dental care for a larger number of individuals with autism.

## 2 MATERIAL AND METHODS

After IRB approval was obtained (IRB ID#202006350), a query was performed in the University of Iowa College of Dentistry and Dental Clinics electronic health records for patients matching three inclusion criteria: being 18 years old or older at the time of first appointment, having self‐reported autism in their health history questionnaires, and had at least one preventive dental procedure been recorded.

De‐identified data was collected from these records and provided by the information technology team member to the researchers in an Excel spreadsheet. The retrieved records included information about each patient's age, gender, body mass index (BMI), mental health, heart disease, xerostomia, diabetes, number of medications, type of preventive procedures, and number of preventive procedures. The following ADA codes were used to typify preventive procedures in this study: D1110 (Prophylaxis—Adult), D1110.1 (Prophylaxis—Adult Collegiate Recall), D1110.3 (Pumice Polish), D1110.4 (Prophy—Adult—No Charge—Freshman Clinic), D1110.5 (Ultrasonic Scaling), D1206 (Fluoride Varnish), D1206.4 (Fluoride varnish—No Charge—Freshman Clinic), D1310 (Nutritional counseling), D1330 (Oral Hygiene Instruction—Complex), D1330.1(Oral Hygiene Instruction/Simple), D4346 (Scaling in presence of gingival inflammation—full mouth), D4355 (Full mouth debridement to enable comp eval and diagnosis), D4910 (Periodontal maintenance), D1354 (Caries arresting med—Silver Diamine Fluoride), D1354.1 (Caries arresting med—Silver Diamine Fluoride‐no cost), and D1355 (Caries prev medicament application‐per tooth—not fluorides).

These variables were chosen among all variables available in the existing database to provide a description of the sample demographics (age and gender), selected health history variables that had been previously linked to autism and/or oral health problems, and information to determine the frequency of preventive treatment. An “undetermined” category was used for participants that had their first dental visit in 2019, 2020, or 2021. Due to the COVID‐19 pandemic, we were not able to tell for some of them if they would return consistently for visits. Univariate and bivariate analyses were performed. Then, two different approaches were used to investigate what factors are associated with regular preventive dental care, as follows.

First, bivariate associations between *having consistent preventive dental visits* (at least one per year) and the covariates of interest were determined. Chi‐square tests (or Fisher's exact tests) were used when analyzing categorical variables and Wilcoxon rank sum tests were used when analyzing continuous variables. No adjustments have been made for multiple comparisons. As no bivariate associations were found, no multivariable modeling was attempted for associations between having consistent dental and the covariates of interest.

Second, bivariate associations between the *total number of preventive dental visits* and the covariates of interest were determined. Spearman correlation tests were used when analyzing continuous covariates and Wilcoxon rank sum (or Kruskal‐Wallis for more than two groups) tests were used when analyzing categorical covariates. Again, no adjustments have been made for multiple comparisons.

To further analyze the associations between *covariates of interest and the total number of dental visits*, Poisson regression was considered. To account for the different lengths of time participants visited the College of Dentistry, an offset was included in the model. The offset is the log of the years since the participant first visited the College of Dentistry. After fitting the full model and testing the residuals, it was clear that overdispersion is present in the data. To account for this overdispersion, a quasipoisson model was fit to the data. The quasipoisson model allows the overdispersion to be estimated and accounted for in the modeling. Variable selection was conducted using backward variable selection. First, all variables that had a *p*‐value < .25 in the bivariate analysis were entered into the starting model. The full model included age at first visit, heart disease, and number of medications. After fitting the full model, variables with the largest *p*‐value that was greater than .05 were removed.

## 3 RESULTS

Summary statistics for the covariates of interest are presented in Table 1. The sample was composed of 119 individuals with an average age of 30.8 years (±12.0). The majority were men (67%) and had Medicaid (58%). Average BMI was very high (42.8 ± 24.7), the prevalence of diabetes and heart disease were 16% and 34%, respectively, and a large proportion of individuals (86%) reported mental health problems. The reported use of tobacco was 16%, alcohol use was 19%, and recreational drugs 6.8%. Dry mouth was reported by 32%, and the average number of medications was 7.2 (±5.5). The average number or preventive dental visits was 7.9 (±10.6) with an average of 1.77 visits per year, and the number of patients with consistent preventive dental visits (at least one visit per year) was 42 (35%).

**TABLE 1:** Summary statistics for the covariates of interest for all subjects

Characteristic	*N* = 119^a^
*Age at first visit*	30.8 (12.0)
*Gender*	
Female	39 (33%)
Male	79 (67%)
Unknown	1
*Insurance*	
Medicaid	69 (58%)
Private insurance	36 (31%)
Self‐pay	13 (11%)
Unknown	1
*BMI*	42.8 (24.7)
Unknown	14
*Diabetes*	18 (16%)
Unknown	4
*Heart disease*	39 (34%)
Unknown	3
*Tobacco use*	19 (16%)
*Alcohol use*	23 (19%)
Unknown	1
*Recreational drugs use*	8 (6.8%)
Unknown	1
*Patients reporting a mental health condition*	96 (86%)
Unknown	7
*Reported dry mouth*	28 (32%)
Unknown	32
*Number of medications*	7.2 (5.5)
*Preventive dental visits*	7.9 (10.6)
*Patients with consistent preventive dental visits*	
Consistent	42 (35%)
Not consistent	43 (36%)
Undetermined	34 (29%)

^a^ Statistics presented: Mean (SD); *n* (%).

Group comparisons were made to determine differences in having *consistent preventive dental visits* and the covariates of interest. An independent *t*‐test revealed no statistically significant associations (Table 2). Similar results were found when checking Spearman rank coefficient between the *total number of preventive dental visits* and the covariates of interest (Table 3), except for a weak positive association with number of medications (Rho = .203; *p*‐value = .027).

**TABLE 2:** Bivariate tests comparing the two groups of interest, consistent visits and nonconsistant visits, for the covariates of interest. Descriptive statistics for the overall sample excluding the participants with undetermined visit status are also provided. The median and first and third quartiles are presented for continuous variables; the frequency and percent are presented for categorical variables

	Overall sample	Consistent visits	Non‐consistent visits	
	*N = 85*	*N = 42*	*N = 43*	*p*‐value
*Age at first visit*	26.0 [22.0;37.1]	26.2 [22.1;40.2]	26.0 [21.2;35.4]	.574
*Gender*				.227
Female	24 (28.6%)	9 (21.4%)	15 (35.7%)	
Male	60 (71.4%)	33 (78.6%)	27 (64.3%)	
*Insurance*				.467
Medicaid	48 (57.1%)	24 (58.5%)	24 (55.8%)	
Private insurance	26 (31.0%)	14 (34.1%)	12 (27.9%)	
Self‐pay	10 (11.9%)	3 (7.32%)	7 (16.3%)	
*BMI*	41.0 [23.0;66.0]	42.0 [23.0;65.0]	40.0 [23.8;66.2]	.878
*Diabetes*				.397
No	70 (85.4%)	36 (90.0%)	34 (81.0%)	
Yes	12 (14.6%)	4 (10.0%)	8 (19.0%)	
*Heart disease*				.169
No	55 (65.5%)	31 (73.8%)	24 (57.1%)	
Yes	29 (34.5%)	11 (26.2%)	18 (42.9%)	
*Tobacco use*				.277
No	70 (82.4%)	37 (88.1%)	33 (76.7%)	
Yes	15 (17.6%)	5 (11.9%)	10 (23.3%)	
*Alcohol use*				>.99
No	65 (76.5%)	32 (76.2%)	33 (76.7%)	
Yes	20 (23.5%)	10 (23.8%)	10 (23.3%)	
*Recreational drugs use*				>.99
No	77 (90.6%)	38 (90.5%)	39 (90.7%)	
Yes	8 (9.41%)	4 (9.52%)	4 (9.30%)	
*Patients reporting a mental health condition*				.348
No	12 (15.0%)	8 (20.0%)	4 (10.0%)	
Yes	68 (85.0%)	32 (80.0%)	36 (90.0%)	
*Reported dry mouth*				.224
No	45 (67.2%)	25 (75.8%)	20 (58.8%)	
Yes	22 (32.8%)	8 (24.2%)	14 (41.2%)	
*Number of medications*	6.00 [3.00;10.0]	6.00 [2.00;11.5]	5.00 [3.00;9.00]	.489

**TABLE 3:** Sperman rank order correlation coefficients (Rho) and *p*‐values (Wilcoxon rank sum or Kruskal‐Wallis); *p*‐values are provided for the association between the total number of preventive dental visits and the continuous and categorical coviariates of interest, respectively

	Rho	*p*‐value
Age at first visit	.124	.180
Gender	NA	.395
Insurance	NA	.443
BMI	.051	.607
Diabetes	NA	.991
Heart disease	NA	.166
Tobacco use	NA	.279
Alcohol use	NA	.546
Recreational drugs use	NA	.519
Patients reporting a mental health condition	NA	.584
Reported dry mouth	NA	.996
Number of medications	.203	.027

NA: not applicable.

In the quasipoisson regression model used to further analyze the associations between covariates of interest and the *total number of preventive dental visits*, the number of medications was the only variable retained in the final model (Table 4). The estimated coefficient suggests taking one additional medication leads to a minimal change of 1.04 (1.01, 1.07) times increase in the rate of dental visits.

**TABLE 4:** Quasipoisson final model used to analyze the associations between covariates of interest and the total number of preventive dental visits

Term	Exponentiated coefficient (95% CI)	Standard error	*p*‐value
Intercept	1.20 (0.90, 1.58)	0.14	.22
Number of medications	1.04 (1.01, 1.07)	0.01	.006

## 4 DISCUSSION

The proportion of adults with autism in this sample who received consistent preventive dental care (a minimum of one visit per year) was only 35%, or only about one in every three patients. The majority of the sample was male (67%), which is consistent with the prevalence of ASD. This result is even direr considering this sample is composed of patients who have had at least one preventive procedure recorded. In a previous study,^11^ from 244 persons with autism getting dental care in a dental school, only about half had received a preventive treatment. One should also note that these samples are from patients seeking dental care, and therefore does not take into account those adults with autism not actively seeking dental care.

Surprisingly, our investigation about possible explanatory variables, which could help to understand what factors are associated with consistent preventive dental visits, showed none of the available variables were associated with *consistent preventive dental visits* in a statistically significant way. Similarly, only *number of medications* was associated with the number of preventive dental visits, and this association was weak. In the quasipoisson regression model used to further analyze the predictors between covariates of interest and the total number of dental visits, the only variable retained in the final model was the number of medications. However, its effect was so small that it cannot be considered clinically significant.

One can assume taking more medications presumably means having more understanding of health needs and therefore more medical care, leading to more frequent dental visits. However, it is also fair to assume an opposing hypothesis, that people who are more medicated may be more ill, and therefore less able to visit the dental office frequently. Nevertheless, it was especially surprising that well‐known enablers such as dental insurance,^15^ or barriers, such as mental health problems,^8^ were not associated with the frequency of preventive dental visits. In part, the lack of statistically or clinically significant associations can be explained by the reduced sample size.

The sample size is a major limitation of this study. Another limitation is the retrospective nature of the investigation and the restrictions imposed by the electronic health record itself, which limited the number of explanatory variables that could be used. Other possible explanatory variables previously reported to be barriers for persons with autism to access dental care, such as caregivers' health and dental literacy, poverty ratio, household education, and non‐English language,^15^ could not be assessed as these variables are absent in the available data. Another limitation to be considered is that these patients might have visited another dental office during the observation period, although we feel that is unlikely since the College of Dentistry is one of the few providers in the state who provide dental care to adults with autism. One reason it is important to expand the sample size beyond the state of Iowa in future studies is because other states have different Medicaid policies.

Therefore, future research to expand this analysis should include larger, national samples using electronic health record consortiums and/or investing more resources into prospective, multicenter or private practice network projects to identify specific barriers and enablers related to accessing preventive dental care for adults with autism.

From the practice management perspective, it is important to highlight that the College of Dentistry has an automated recall system that sends recall cards for the patients and gives robot calls confirming patients’ appointment a day before it is scheduled. Missing appointments are usually followed‐up by the provider and front desk team. Although, this management practice seems appropriate, the results of this study show the need for a careful review of the follow‐up system.

## 5 CONCLUSION

In this sample of adults with autism receiving preventive care in a dental school, only about one in every three adults with autism has received at least one preventive dental procedure per year. No significant barriers or enablers were found among the available explanatory variables.

## CONFLICTS OF INTEREST

The authors declare no conflicts of interest.

## ETHICS STATEMENT

The authors declare that the study conforms to recognized ethical standards and was approved by the University of Iowa Institutional Review Board (IRB ID#202006350).
